# Imaging the accumulation and suppression of tau pathology using multiparametric MRI

**DOI:** 10.1016/j.neurobiolaging.2015.12.001

**Published:** 2016-03

**Authors:** Holly E. Holmes, Niall Colgan, Ozama Ismail, Da Ma, Nick M. Powell, James M. O'Callaghan, Ian F. Harrison, Ross A. Johnson, Tracey K. Murray, Zeshan Ahmed, Morton Heggenes, Alice Fisher, M.J. Cardoso, Marc Modat, Simon Walker-Samuel, Elizabeth M.C. Fisher, Sebastien Ourselin, Michael J. O'Neill, Jack A. Wells, Emily C. Collins, Mark F. Lythgoe

**Affiliations:** a Division of Medicine, Centre for Advanced Biomedical Imaging, University College London, London, UK; b Translational Imaging Group, Centre for Medical Image Computing, University College London, London, UK; c Eli Lilly & Co. Ltd, Windlesham, Surrey, UK; d Tailored Therapeutics, Eli Lilly and Company, Lilly Corporate Center, Indianapolis, IN, USA; e Department of Neurodegenerative Diseases, Institute of Neurology, University College London, London, UK

**Keywords:** Alzheimer's disease, MRI, Biomarker, Longitudinal, Tauopathy, In vivo

## Abstract

Mouse models of Alzheimer's disease have served as valuable tools for investigating pathogenic mechanisms relating to neurodegeneration, including tau-mediated and neurofibrillary tangle pathology—a major hallmark of the disease. In this work, we have used multiparametric magnetic resonance imaging (MRI) in a longitudinal study of neurodegeneration in the rTg4510 mouse model of tauopathy, a subset of which were treated with doxycycline at different time points to suppress the tau transgene. Using this paradigm, we investigated the sensitivity of multiparametric MRI to both the accumulation and suppression of pathologic tau. Tau-related atrophy was discernible from 5.5 months within the cortex and hippocampus. We observed markedly less atrophy in the treated rTg4510 mice, which was enhanced after doxycycline intervention from 3.5 months. We also observed differences in amide proton transfer, cerebral blood flow, and diffusion tensor imaging parameters in the rTg4510 mice, which were significantly less altered after doxycycline treatment. We propose that these non-invasive MRI techniques offer insight into pathologic mechanisms underpinning Alzheimer's disease that may be important when evaluating emerging therapeutics targeting one of more of these processes.

## 1 Introduction

It has been over a century since Alois Alzheimer first described the symptoms of the presenile dementia that would come to bear his name (Stelzmann et al., 1995); but to date, there is still no disease-modifying or preventative treatment for this devastating disease. As the incidence of Alzheimer's disease (AD) continues to rise to epidemic proportions (Brookmeyer et al., 2007), effective therapies are urgently required to ease both the economic and emotional burdens of this devastating disease.

The two key neuropathologic hallmarks of AD are plaques comprised of amyloid-beta (Aβ) peptides and neurofibrillary tangles (NFTs) of hyperphosphorylated tau. Emerging therapies targeting the production or clearance of these protein aggregates require robust biomarkers to evaluate and quantify therapeutic efficacy. The development of reliable biomarkers in humans, however, is complicated by the length of the clinical studies required to follow the evolution of pathology; this is currently believed to occur as much as 30–40 years before cognitive deficits (Jack et al., 2013). One approach is to establish and validate biomarkers using mouse models of AD as a surrogate for patient populations.

Transgenic mouse models of AD have served as valuable tools for investigating pathogenic mechanisms relating to neurodegeneration. Mice containing mutations in the amyloid precursor protein (APP) gene are the oldest and most widely studied models of AD and are used to investigate the role of APP, Aβ, and amyloidosis in neurodegeneration (Hall and Roberson, 2012). Despite compelling evidence suggesting that Aβ may be the primary cause of AD, the therapeutic efficacy of Aβ immunization observed in animal models of AD has yet to be translated successfully in patients with AD (Giacobini and Gold, 2013). Tau pathology, not Aβ burden, independently predicts cognitive status in patients with AD (Giannakopoulos et al., 2003). This suggests the potential value of tau-targeted therapies in AD and the requirement for non-invasive biomarkers that are sensitive to the severity of tau pathology.

To investigate tau-specific biomarkers of pathology, we have used the tetracycline operator-MAPT*P301L (or rTg4510) mouse model (Ramsden et al., 2005), which expresses a repressible form of the human P301L mutant of the 4-repeat microtubule-associated protein tau (MAPT) gene, under the control of a tetracycline-responsive element. The rTg4510 mouse develops robust NFT pathology in the forebrain around 4 months of age, with the cortex and hippocampus being severely affected (SantaCruz et al., 2005), a distribution similar to that seen in AD patients (Braak and Braak, 1995). One advantage of the model is that the expression of mutant tau can be effectively suppressed by the administration of the tetracycline derivative doxycycline (SantaCruz et al., 2005). Longitudinal assessment of this model enables the investigation of biomarkers sensitive to the accumulation of tau pathology and provides a framework to evaluate the efficacy of therapeutic strategies targeted at the suppression or removal of pathologic tau.

In this work, we sought to identify specific elements of the pathologic cascade and the advancement and inhibition of tau pathology, using multiparametric magnetic resonance imaging (MRI) measures in the rTg4510 mouse. We explored morphologic changes using high-resolution structural MRI, the breakdown in chemical exchange of mobile proteins using amide proton transfer (APT) imaging, microstructural changes in the cytoarchitecture through diffusion tensor imaging (DTI), and alterations in cerebral blood flow (CBF) using arterial spin labelling (ASL), over time.

The rTg4510 mouse accumulates an early burden of tau pathology, which progresses rapidly (SantaCruz et al., 2005). Pretangles, a premature state to NFTs, have been observed from 2.5 months, followed by mature tangle formations between 3 and 5.5 months of age (Yue et al., 2011). To coincide with the progressive and dynamic accumulation of tau pathology in this model, we conducted 2 longitudinal studies: one group received doxycycline from 3.5 months and the second cohort received doxycycline from 4.5 months. Using these multiparametric MRI techniques, we demonstrate sensitivity to tau-driven pathologic changes and tau suppression in the rTg4510 in mice treated at 2 distinct time points.

## 2 Materials and methods

### 2.1 Animals

The rTg4510 animal model used in this study has been previously characterized and described in the literature, predominantly by using histologic techniques (SantaCruz et al., 2005). Female rTg4510 mice and litter-matched wild-type controls were bred on a mixed FVB/NCrl + 129S6/SvEvTa background for Eli Lilly and Company by Taconic (Germantown, MD, USA) and received on site 2 weeks before the initiation of the studies.

**Supplementary Fig. 1:**
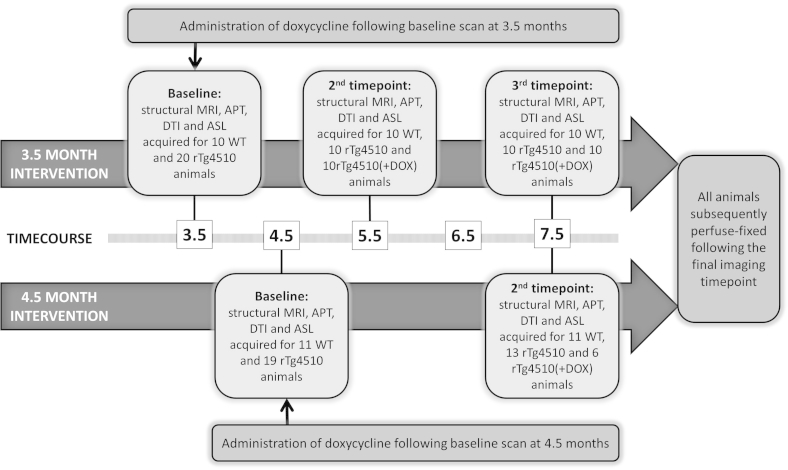
Schematic describing the 3.5- and 4.5-month doxycycline intervention studies. High-resolution structural magnetic resonance imaging (MRI), amide proton transfer (APT), diffusion tensor imaging (DTI), and arterial spin labelling (ASL) were acquired at all time points. Doxycycline was administered to a subset of rTg4510 animals after the baseline scan; all treated rTg4510 animals were maintained on a doxycycline mixed chow diet until the end of each respective study.

Two doxycycline intervention cohorts are reported in this study and summarized in Supplementary Fig. 1. The 3.5-month intervention cohort consisted of 20 rTg4510 mice with 10 wild-type controls, and the 4.5-month intervention cohort consisted of 19 rTg4510 mice with 11 wild-type controls. Subsequent to their initial scan, the rTg4510 mice were divided into 2 subgroups: the treated rTg4510 mice received 2 boluses of 10 mg/kg doxycycline hyclate (Sigma Aldrich, 10 mg per kg of body weight) via oral gavage and were maintained on doxycycline-mixed chow diet (Harlan Teklad Rodent Diet, 200 mg doxycycline per kg of dietary chow) until the end of the study and the untreated rTg4510 mice and the wild-type controls received 2 boluses of a 5% glucose vehicle via oral gavage and were maintained on standard chow. The dietary content of the doxycycline-mixed and standard chow was consistent for each group.

For further evaluation of the rTg4510 mouse's response to increasing concentrations of isoflurane, a separate cohort of female rTg4510 mice and wild-type controls were imaged at 9 months.

All mice were kept in standard size mouse cages (29 × 18 × 13 cm, up to 5 per same sex groups) at 20–26 °C on a daily 12-hour light-dark cycle with ad libitum access to food and water. All studies were carried out in accordance with the UK Animals (Scientific Procedures) Act of 1986 and subject to approval by the internal ethical review panel of University College London.

### 2.2 Magnetic resonance imaging

All imaging was performed with a 9.4-T VNMRS horizontal bore scanner (Agilent Inc). A 72-mm inner diameter volume coil (Rapid Biomedical) was used for radiofrequency transmission, and signal was received using a 4-channel array head coil (Rapid Biomedical).

Mice were placed in an induction box before anesthesia was induced using 2% isoflurane at 1 L per minute in 100% O_2._ Mice were subsequently positioned in an MRI-compatible head holder to minimize motion artifacts. Anesthesia was maintained throughout imaging using 1.5% (±0.2%) isoflurane at 1 L per minute in 100% O_2_ delivered via a nose cone, which permitted spontaneous breathing of the mice. Core temperature and respiratory rate were monitored using a rectal probe and pressure pad, respectively (SA Instruments). Mice were maintained at ∼37 °C using heated water tubing and a warm air blower with a feedback system (SA Instruments). Respiration rate was maintained between 80 and 120 breaths per minute by manually adjusting the isoflurane vaporizer. Scans were performed in the following order: chemical exchange saturation transfer (CEST), ASL, DTI, and structural imaging. Before imaging, shimming was first performed across a volume specifically confined to the CEST imaging slice (3 mm slice thickness [coronal]). After CEST acquisition, shimming was performed across the whole mouse brain for the structural, ASL, and DTI sequences.

Mice within the 3.5-month intervention cohort were scanned at baseline (3.5 months) and subsequently at 5.5 and 7.5 months. Mice within the 4.5-month intervention cohort were scanned at baseline (4.5 months) and subsequently at 7.5 months. In both studies, administration of doxycycline was commenced after the baseline scan. All animals were perfuse fixed after the final imaging time point at 7.5 months before histologic evaluation (see Section 2.7). We employed high-resolution structural MRI, CEST, DTI, and ASL at each time point to characterize the treated and untreated rTg4510 mice and wild-type controls.

### 2.3 Structural imaging

A 3-dimensional T2-weighted fast spin echo sequence was employed for structural imaging with the following parameters: field of view (FOV) = 19.2 × 16.8 × 12.0 mm, resolution = 150 × 150 × 150 μm, repetition time (TR) = 2500 ms, effective echo time = 43 ms, echo train length = 4, number of signal averages = 1, and imaging time = 1.5 hours.

#### 2.3.1 Processing for tensor-based morphometry

At each time point, the in vivo structural images were automatically oriented to a standard atlas space (right anterosuperior), corrected for intensity nonuniformity using the N4ITK algorithm (Tustison et al., 2010), and skull stripped using a label fusion algorithm, STEPS (Jorge Cardoso et al., 2013), combining masks from several prior atlases registered to the data. Intensities were normalized (Nyul et al., 2000), and a multi-iteration group-wise registration (implemented in the open-source NiftyReg software (Modat et al., 2010) was performed as follows to align equivalent voxels between subjects. First, all subjects were rigidly aligned to a randomly chosen target member of the group. This was followed by 4 iterations of affine registration (12° of freedom), using a block-matching algorithm to optimize normalized mutual information and 15 iterations of non-rigid registration, based on symmetric free-form deformation (Modat et al., 2012). After each iteration, the intensity average image was calculated and used as the target for the subsequent registrations. The determinant of the Jacobian matrix was calculated at each voxel of the resulting deformation fields, giving that voxel's relative expansion or contraction in the space of the final average. These values were smoothed with a 0.2-mm full-width half-maximum Gaussian kernel to account for registration error and log transformed. Mass-univariate statistics (2-tailed *t* tests) were performed at each voxel, fitting a general linear model to compare groups. The resulting statistical parametric maps were corrected for multiple tests using the false discovery rate (Genovese et al., 2002, *q* = 0.05).

#### 2.3.2 Automatic structural parcellation

Using a multiatlas-based structural parcellation framework (Ma et al., 2014), we extracted 3 structures of interest: the cortex, hippocampus, and thalamus for each animal at each time point. The brain images were oriented, nonuniformity corrected, and skull stripped, as earlier. We adopted the publicly available in vivo mouse brain MRI atlas previously published by Ma et al. (2008) for the framework. First, the atlas images were registered affinely to the original MRI image data using a block-matching algorithm (Ourselin et al., 2000). Once complete, the STAPLE algorithm (Ma et al., 2014) was applied to fuse the resampled atlas masks together to create a consensus brain mask for each animal's scans. A further non-rigid registration based on fast free-form deformation was then performed to correct any remaining local misalignment of the affinely registered atlas to the brain volumes (Modat et al., 2010). The structural labels from the atlas were then resampled to match the resolution of the brain scans and fused using the STEPS algorithm (Jorge Cardoso et al., 2013) to create the final parcellated structures of interest: the cortex, hippocampus, and thalamus. A previously published calibration protocol was used to correct gradient scaling errors in the data (O’Callaghan et al., 2014). Absolute volumes were normalized to the total brain volume to calculate the normalized volume change. Two-way analysis of variance *t* tests were applied to volumes of structures of interest, for each animal in each group at its corresponding time point. The Tukey range test was applied to correct for multiple comparisons.

### 2.4 Chemical exchange saturation transfer

The CEST sequence was acquired using a single slice gradient echo imaging sequence positioned axial using the splenium of the corpus callosum as a landmark for consistency of slice positioning between subjects (TR = 6.1 ms, echo time = 2 ms, flip = 5°, FOV = 20 × 20 mm, slice thickness = 3 mm, matrix size = 64 × 64). Saturation pulses were applied at 79 frequency offsets covering ±6 ppm to encompass APT saturation peaks around +3.5 ppm. A reference offset at 200 ppm was also acquired for normalization. APT was calculated as the area under the MTRasym curves between 3.3 and 3.7 ppm on a pixel-by-pixel basis by fitting a polynomial function to Z spectra, correcting for off-resonance effects by cubic spline interpolation and subtracting the signal intensities at either side of the direct water saturation peak (van Zijl and Yadav, 2011). Total imaging time was 5 minutes.

### 2.5 Diffusion tensor imaging

A 4-shot spin echo echo planar imaging sequence was used to acquire 16 slices. The fissure between the olfactory bulbs and the cortex was used as an anatomic landmark to maintain consistency in slice positioning between animals. The FOV was 20 × 20 mm with a matrix size of 100 × 100 and a slice thickness of 0.5 mm. Diffusion gradients were applied in 20 directions with the following parameters *G* = 0.25 T/m, Δ = 9.3 ms, δ = 5.5 ms, and *b* = 1050 seconds per mm^2^ to generate diffusion-weighted images in addition to a single unweighted B0 image. Acquisition of 5 averages with a TR of 2000 ms gave a total imaging time of 30 minutes. Software written in Matlab was used to construct tensors at each voxel through a least-squares solution approach (Batchelor et al., 2003). The parameters fractional anisotrophy (FA), mean diffusivity (MD), and radial diffusivity (RD) were calculated from the tensors following standard methods (Le Bihan et al, 2001, Song et al, 2002). Total imaging time was 25 minutes.

### 2.6 Arterial spin labelling

A flow-sensitive alternating inversion recovery sequence (Kim, 1995, Kwong et al, 1992) with a 4-shot segmented spin echo echo planar imaging readout was implemented with the following parameters: 5 slices, slice thickness = 1 mm, FOV = 20 × 20 mm, matrix size = 64 × 64, slice selective inversion pulse width = 12 mm, inversion time = 1500 ms, echo time = 11 ms, TR = 3500 ms, and 20 averages. A hyperbolic secant adiabatic inversion pulse was used with a bandwidth of 20 kHz for the flow-sensitive alternating inversion recovery labelling pulses (Wells et al., 2012). The splenium of the corpus callosum was used as a landmark for consistency of slice positioning between subjects. Total imaging time was 20 minutes. CBF maps were generated using the model described by Buxton et al. (1998).

### 2.7 Perfuse fixation

After in vivo imaging, mice were terminally anesthetized with euthanal (0.1 mL) administered via intraperitoneal injection. The thoracic cavities were opened and the mice perfused through the left ventricle with 15–20 mL of saline (0.9%) followed by 50 mL of buffered formal saline at a flow rate of 3 mL per minute. After perfusion, the animal was decapitated, defleshed, and the lower jaw removed. All brains were stored in-skull at 4 °C before being dispatched for histology.

### 2.8 Histology and immunohistochemistry

Brain samples were processed using the Tissue TEK VIP processor (GMI Inc, Ramsey, MN, USA) before being embedded in paraffin wax for coronal brain sectioning. Serial sections (6 μm) were taken using HM 200 and HM 355 (Thermo Scientific Microm, Germany) rotary microtomes. Immunohistochemistry (IHC) was performed using a primary antibody for tau phosphorylated at serine 409 (PG-5, 1:500 from Peter Davies; Albert Einstein College of Medicine, Bronx, NY, USA) as previously described (Ahmed et al., 2014). Stained sections were digitized using the Scanscope XT slide scanner (Aperio, Vista, CA, USA) at ×20 magnification. Imagescope software (version 11.1.2.760; Aperio) was used to view the digitized tissue sections and delineate the regions of interest (ROIs) (cortex, hippocampus, and thalamus). The number of PG-5–positive neurons was manually counted within a delineated region and expressed as a density (mm^2^). Both sides of the brain were analyzed and averaged before statistical analysis.

### 2.9 Statistical analysis

**Supplementary Fig. 2:**
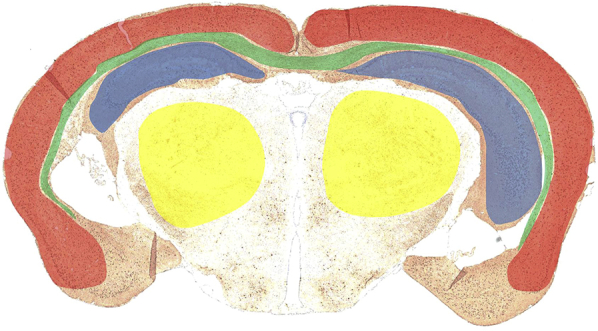
Coronal histology slice from a representative rTg4510 animal, showing location of hippocampal (blue), cortical (red), thalamic (yellow), and white matter (green) regions of interest for quantitative multiparametric magnetic resonance imaging analysis. The cortical and hippocampal results were averaged to extract parameters within the “high-ranked” tau pathology regions.

For the structural parcellation analysis, the volumes of the cortex, hippocampus, and thalamus represent the whole respective structures. For the CEST, DTI, and ASL analysis, measurements were taken from ROIs drawn in a single slice positioned approximately bregma –2.3; this was to align our MRI measurements with the coronal section that was used to quantify NFT burden in our histologic analysis. ROIs were manually drawn in the cortex, hippocampus, thalamus, and corpus callosum (DTI only), as illustrated in Supplementary Fig. 2. The results within the hippocampus and cortex were averaged to calculate parameters within regions suffering from “high” levels of tau pathology.

Quantitative data were analyzed using Student *t* test for pairwise comparisons and 2-way analysis of variance, followed by Sidak post hoc tests for group-wise comparisons. GraphPad Prism 5 (version 5.04; GraphPad Software Inc, La Jolla, CA, USA) was used to perform statistical tests and to produce graphs, which display mean values ± standard deviation (SD). Statistical significance was set at *p* ≤ .05.

## 3 Results

In this work, we have explored the sensitivity of multiparametric MRI to the accumulation and suppression of pathologic tau. A detailed description of the imaging protocol is provided in Supplementary Fig. 1, including the 3.5- and 4.5-month intervention strategies.

### 3.1 Histology and IHC

**Fig. 1:**
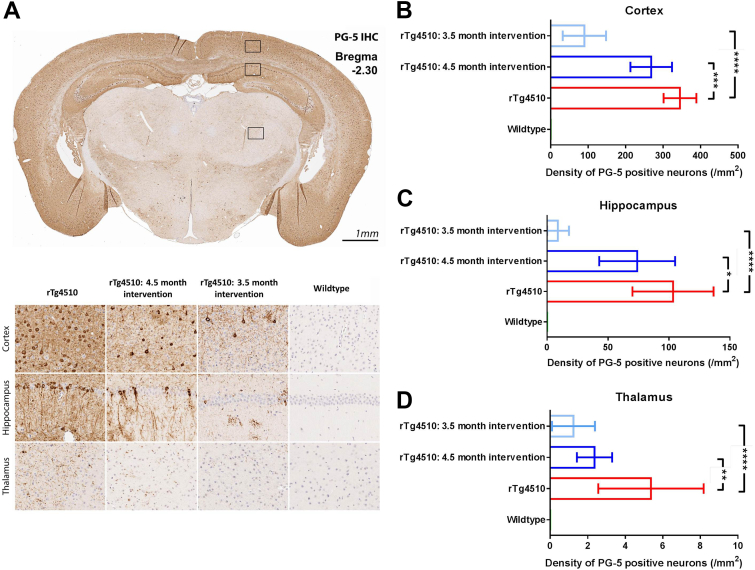
Immunohistochemistry to estimate regional tau phosphorylated at serine 409 (PG-5)–positive neurofibrillary tangle (NFT) density. (A) Representative coronal slice illustrating the distribution of PG-5 positive NFTs in the untreated rTg4510s, with visible regional specificity in treated and untreated animals. Quantitative regional estimates of cortical (B), hippocampal (C), and thalamic (D) NFT density for each of the wild-type (*n* = 21), untreated rTg4510s (*n* = 20), 4.5 months intervention Tg4510s (*n* = 6), and 3.5 months intervention rTg4510s (*n* = 10) at 7.5 months of age. The y axis has been independently adjusted for each region under investigation to account for the marked range in PG-5 density. Error bars represent the standard deviation. **p ≤ .01 and ****p ≤ .0001.

To quantify the severity of tau pathology in the doxycycline-treated and untreated rTg4510 groups, all mice (*n* = 21 wild-type, 23 untreated, 10 rTg4510 [3.5 months intervention] and 6 rTg4510 [4.5 months intervention]) were sacrificed after the final imaging time point (7.5 months) and prepared for neuropathologic assessment. IHC using PG-5 (pS409), a marker of tau hyperphosphorylation, was used to quantify the density of tau-positive neurons in the cortex, hippocampus, and thalamus (Fig. 1A); the results can be seen in Fig. 1. Consistent with the literature (SantaCruz et al., 2005), untreated rTg4510 mice contained a high density of PG-5 positive neurons in the cortex and hippocampus (mean = 345.2 ± 43.8 and 103.3 ± 33.3 [SD], respectively, Fig. 1A–C). In contrast, the thalamus had a significantly lower density (mean = 5.34 ± 2.8 [SD], Fig. 1A and D). For this reason, we classified the cortex and hippocampus as “high tau burden” regions and the thalamus as a “low tau burden” region. There was no PG-5–positive staining observed in the wild-type mice. (Fig. 1B–D).

After administration of doxycycline to rTg4510 mice, we observed a significant reduction in the number of PG-5–positive neurons in the cortex, hippocampus, and thalamus of the 3.5-month intervention (*p* < .0001) and 4.5-month intervention (*p* < .001, *p* < .05, and *p* < .01, respectively) mice compared with the untreated group (Fig. 1A–D). The group treated with doxycycline from 3.5 months showed the greatest reduction in PG-5–positive neuron density, especially in the hippocampus and thalamus regions, illustrating the effectiveness of earlier intervention in this model.

### 3.2 High-resolution structural MRI

**Fig. 2:**
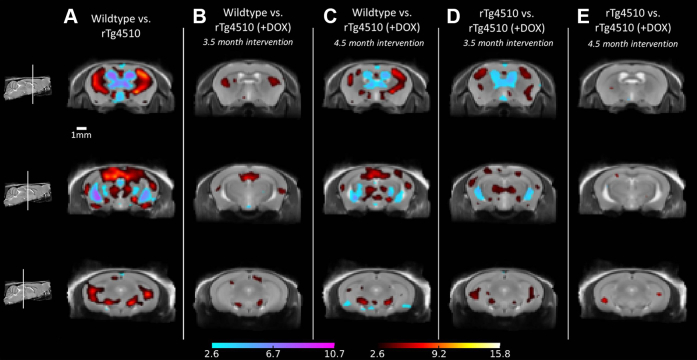
Structural analysis at 7.5 months, showing tensor-based morphometry statistical results overlaid on representative coronal slices of the final group average images after 15 iterations of non-rigid registration. Red: regions where the rTg4510 brains are relatively locally smaller than the average and blue: rTg4510 brains are locally larger. Based on false discovery rate–corrected *t* statistics (*q* = 0.05). (For interpretation of the references to color in this figure legend, the reader is referred to the Web version of this article.)

To investigate morphometric changes in the rTg4510 mice, we acquired high-resolution in vivo 3-dimensional structural images for the 2 longitudinal cohorts. We employed tensor-based morphometry (TBM) to identify local areas of significant brain atrophy or expansion; the results at 7.5 months are shown in Fig. 2. For each set of comparisons, we present 3 coronal slices with TBM statistics overlaid. Extensive bilateral morphometric changes were detected in the untreated rTg4510 mice compared with wild-type controls, including significant atrophy in the forebrain, cortex, lateral striata, and hippocampus and expansion of the lateral, third, and fourth ventricles (Fig. 2A). Reductions were also observed in the thalamus; in the absence tau pathology in this region, this is likely to reflect global differences in brain volume of the rTg4510 mice compared with wild-type controls.

To investigate the morphologic changes within the 2 doxycycline-treated rTg4510 mice cohorts, we made 2 comparisons: first, wild-type versus doxycycline-treated rTg4510 mice (Fig. 2B and C), and second, untreated rTg4510 versus the treated rTg4510 mice (Fig. 2D and E). In rTg4510 mice treated with doxycycline from 3.5 months, we observed small discrete structural changes within the caudate putamen and the hippocampus compared with wild-type controls (Fig. 2B), indicating preservation of cortical regions and ventricular spaces that presented with markedly fewer significant voxels. Figure 2D compares mice treated with doxycycline from 3.5 months with untreated rTg4510 mice. Here, TBM detected gross relative expansion of the ventricles and a bilateral pattern of cortical atrophy, highlighting regions preserved because of suppression of the tau transgene. After administration of doxycycline from 4.5 months, a similar yet more marked pattern of changes was observed in comparison with wild-type controls (Fig. 2C). The comparison between rTg4510 mice treated with doxycycline from 4.5 months and untreated rTg4510 mice revealed a small number of significant voxels within the hippocampus, indicating minor differences between these 2 cohorts (Fig. 2E); the earlier (3.5 months) intervention appeared more successful at limiting atrophy and ventricular expansion.

**Supplementary Fig. 3:**
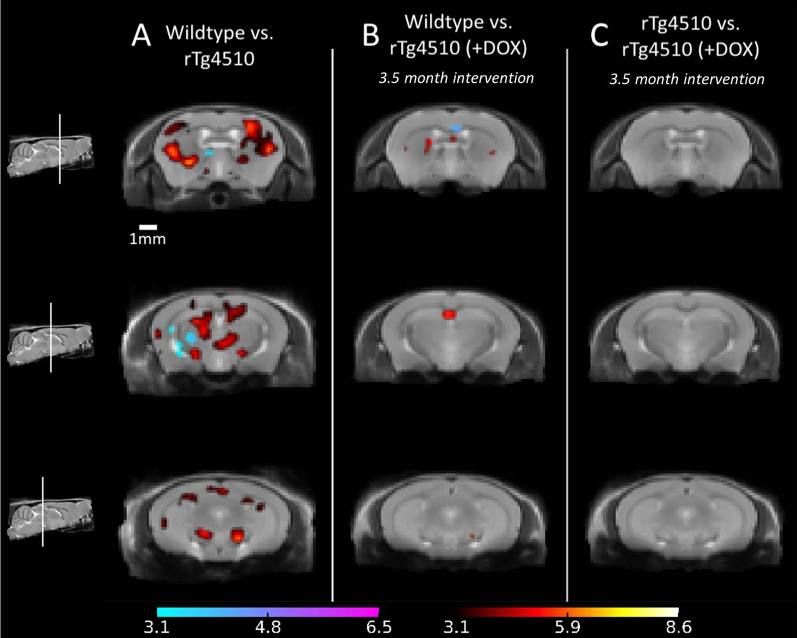
Results from structural analysis at 5.5 months, showing tensor-based morphometry statistical results overlaid on representative coronal slices of the final group average image after 15 iterations of nonrigid registration. Red: regions where the rTg4510 brains are relatively locally smaller than the average and blue: rTg4510 brains are locally larger. Based on false discovery rate–corrected *t* statistics (*q* = 0.05).

At 5.5 months (Supplementary Fig. 3B), TBM detected comparable yet less widespread differences between the wild-type and rTg4510 mice treated with doxycycline from 3.5 months in the same regions implicated in Fig. 2. In the comparison between the untreated rTg4510 and rTg4510 mice treated with doxycycline from 3.5 months (Supplementary Fig. 3C), no significant voxels survived false discovery rate correction. TBM results at the baseline scan revealed negligible significant local differences between the rTg4510 mice and wild-type controls (data not shown).

To quantify longitudinal volume differences between the rTg4510 mice and wild-type controls in the regions selectively vulnerable to high tau accumulation, we also employed a fully automated atlas-based brain segmentation pipeline to measure absolute and normalized volume changes in the high-ranked pathology regions across all time points. Normalized volume changes were calculated from the regional volume changes divided by the change in the whole brain volume (to account for changes in whole brain volume).

**Fig. 3:**
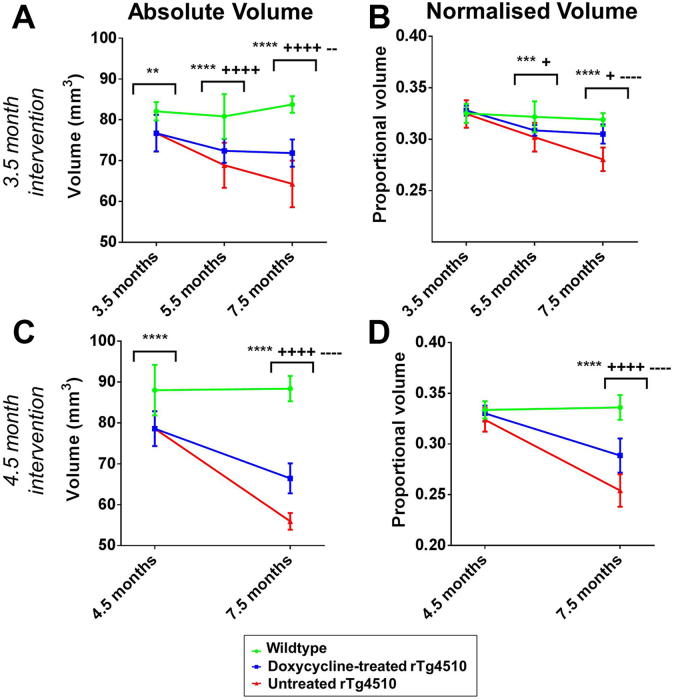
Longitudinal volumetric changes, extracted from the high-resolution structural images. Hippocampal and cortical volumes were averaged to extract absolute and normalized volume changes in the high-ranked pathology regions. We present the absolute volume changes within the high-ranked pathology regions after (A) 3.5 months and (C) 4.5 months intervention. In addition, we normalized the absolute volume changes to the total brain volume to extract the normalized volume changes after (B) 3.5 months and (D) 4.5 months intervention. Statistically significant groups have been identified and highlighted. Error bars represent the standard deviation. Wild-type versus untreated rTg4510s: **p ≤ .01, ***p < .001, and ****p ≤ .0001. Wild-type versus treated rTg4510s: +*p* ≤ .05 and ++++*p* ≤ .0001. Treated rTg4510s versus untreated rTg4510s: −−*p* ≤ .01 and −−−−*p* ≤ .0001.

We detected a significant reduction in absolute volume of the high-ranked pathology regions within the untreated rTg4510 mice from 3.5 months of age compared with the wild-type controls (*p* < .001) (Fig. 3A). The absolute volume of the high-ranked pathology regions within the untreated rTg4510 mice continued to decline by a further 16.2% until the final imaging time point at 7.5 months (Fig. 3A). The rTg4510 mice treated with doxycycline from 3.5 months suffered less atrophy compared with the untreated rTg4510 mice; the decline in volume was reduced to 6.3% owing to the suppression of the transgene with doxycycline (Fig. 3A). Meanwhile, the absolute volume of the wild-type controls remained consistent from 3.5 months (82.1 mm^3^) to 7.5 months (83.8 mm^3^) indicating that the volume changes were specific to the rTg4510 mice (Fig. 3A). A similar trend was observed within the untreated rTg4510 mice in the 4.5-month intervention study (Fig. 3C).

**Supplementary Fig. 4:**
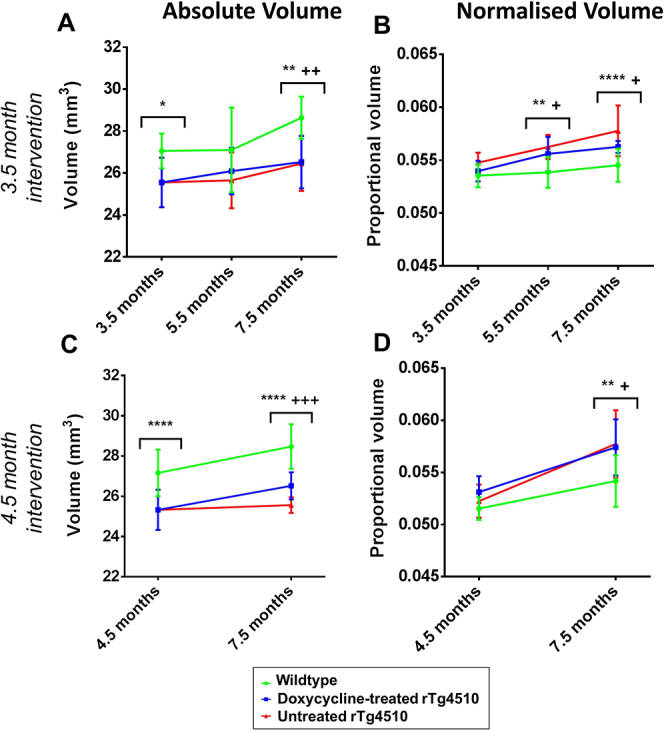
Longitudinal volume changes, extracted from the high-resolution structural images, after (A and B) 3.5 months and (C and D) 4.5 months doxycycline treatment. Results are shown for (A and C) absolute volume and (B and D) normalized volume in the thalamus. Statistically significant groups have been identified and highlighted. Error bars represent the standard deviation. Wild-type versus untreated rTg4510s: *p ≤ .05, **p ≤ .01, and ****p ≤ .0001. Wild-type versus treated rTg4510s: +*p* ≤ .05, ++*p* ≤ .01, and +++*p* ≤ .001.

We observed that the volume of the thalamus, a region of low tau burden, was reduced in the rTg4510 mice compared with wild-type controls from 3.5 months (Supplementary Fig. 4A); these structural changes were similar to the volume reduction in the high tau burden regions (Fig. 3A and C). This result, in the absence of tau pathology, may indicate the effect of the transgene expression on brain morphology, resulting in volume reduction in regions unaffected by tau pathology (Supplementary Fig. 4A and C).

To uncover tau-specific volume changes and remove the confounding effects of the transgene expression, we also explored the normalized volume changes. After normalization of the absolute volumes to total brain volume at each time point, we detected significant volume loss in the high ranked pathology regions of the untreated rTg4510 mice compared with wild-type controls at 7.5 months (*p* < .0001) (Fig. 3B and D). Atrophy was detectable from 5.5 months within these mice (*p* < .001) illustrating the sensitivity of high-resolution structural MRI to tau-specific morphologic changes occurring in the rTg4510 mice (Fig. 3B).

After treatment with doxycycline, we observed significantly less atrophy in the treated rTg4510 mice compared with the untreated rTg4510 mice at 7.5 months (*p* < .0001), after both 3.5 and 4.5 months doxycycline intervention (Fig. 3B and D). Interestingly, the atrophy within the mice treated from 3.5 months was significantly less than rTg4510 mice treated from 4.5 months: 7.0% and 12.4% volume loss, respectively. These results suggest that earlier intervention was able to reduce atrophy to a greater extent.

Normalization of the thalamus to total brain volume resulted in an expected (as this region suffers little tau pathology) increase in normalized thalamic volume within the rTg4510 mice compared with the wild-type controls (Supplementary Fig. 4B and D). This is because of normalization with the decreasing total brain volume, leading to an apparent increase in thalamic volume over time.

### 3.3 Chemical exchange saturation transfer

**Supplementary Fig. 5:**
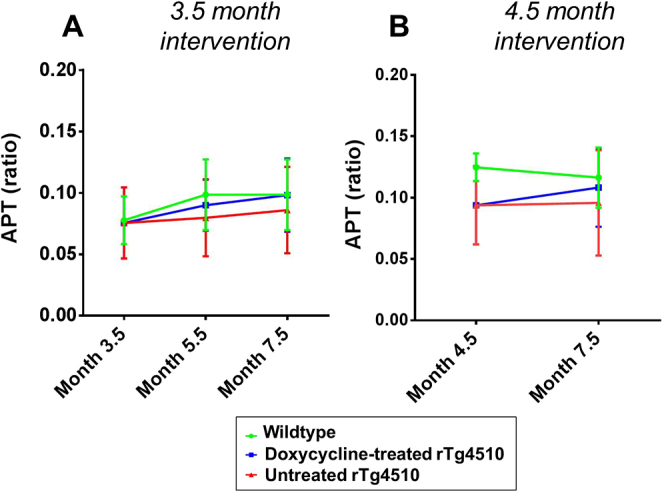
Longitudinal amide proton transfer (APT) results from the thalamus after (A) 3.5 months and (B) 4.5 months doxycycline intervention. No statistically different groups were identified. Error bars represent the standard deviation.

**Fig. 4:**
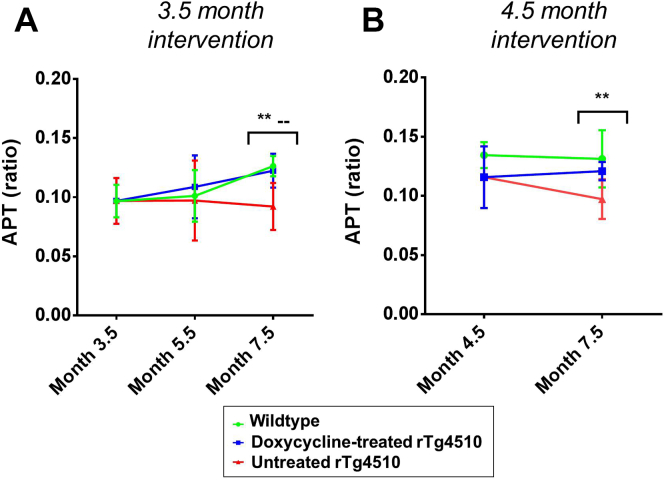
Longitudinal amide proton transfer (APT) results from the high-ranked pathology regions after (A) 3.5 months and (B) 4.5 months doxycycline intervention. Statistically significant groups have been identified and highlighted. Error bars represent the standard deviation. Wild-type versus untreated rTg4510s: *p ≤ .05 and **p ≤ .01. Treated rTg4510s versus untreated rTg4510s: −−*p* ≤ .01.

Figure 4A shows the CEST results within the high-ranked pathology regions of the rTg4510 mice after doxycycline intervention from 3.5 months. Decreased APT was identified in the untreated rTg4510 mice relative to the wild-type controls at 7.5 months (*p* < .01). Within the thalamus, in the presence of low tau burden, APT was not significantly different between the rTg4510 animals and wild-type controls (Supplementary Fig. 5). These results provide evidence in support of APT's sensitivity to tau-related pathology at later time points.

Administration of doxycycline, after both 3.5 and 4.5 months intervention, resulted in an increase in APT toward wild-type values by 7.5 months. At this time point, APT in the treated rTg4510 mice was not significantly different from the wild-type controls (Fig. 4A and B). The rTg4510 mice treated with doxycycline from 3.5 months showed greater conservation: at this time point, both the treated rTg4510 mice and the wild-type controls were significantly different from the untreated rTg4510 mice (*p* < .01) (Fig. 4A). This indicates that the APT signal may be sensitive to the suppression of pathologic conformations of tau.

### 3.4 Diffusion tensor imaging

**Fig. 5:**
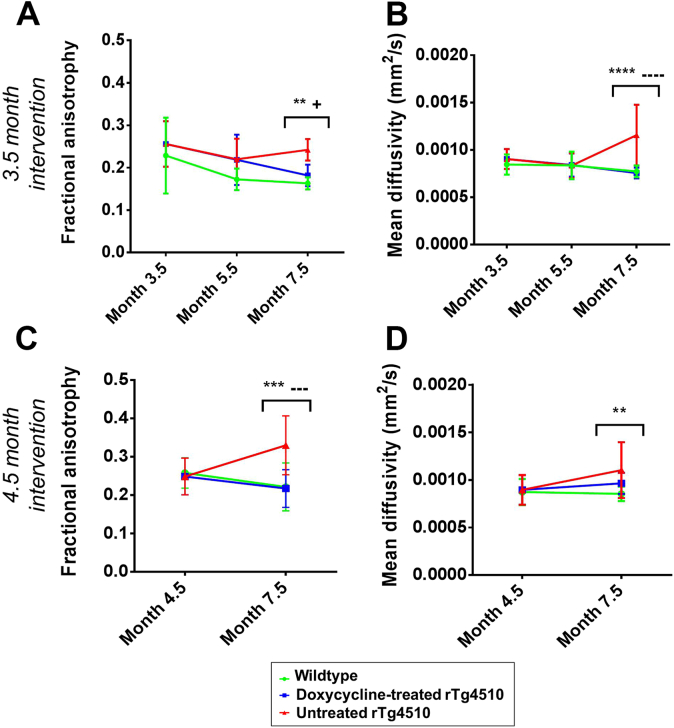
Longitudinal (A and C) fractional anisotropy and (B and D) mean diffusivity in the high-ranked pathology regions after (A and B) 3.5 months and (C and D) 4.5 months doxycycline intervention. Statistically significant groups have been identified and highlighted. Error bars represent the standard deviation. Wild-type versus untreated rTg4510s: **p ≤ .01, ***p ≤ .001, and ****p ≤ .0001. Wild-type versus treated rTg4510s: +*p* ≤ .05. Treated rTg4510s versus untreated rTg4510s: −−−*p* ≤ .001 and −−−−*p* ≤ .0001.

DTI was employed to longitudinally investigate the microstructural changes in the gray and white matter of the rTg4510 mice. The results within the high-ranked pathologic gray matter tissues are shown in Fig. 5. At 7.5 months, the untreated rTg4510 mice exhibited increased FA and MD, with both parameters able to discriminate between the untreated rTg4510 mice and wild-type controls (Fig. 5A–D). Interestingly, at earlier time points, neither FA (Fig. 5A and B) nor MD (Fig. 5C and D) were able to distinguish between the rTg4510 mice and wild-type controls, suggesting that detectable changes in these DTI parameters occur downstream from the formation of tau lesions from 4 months.

**Supplementary Fig. 6:**
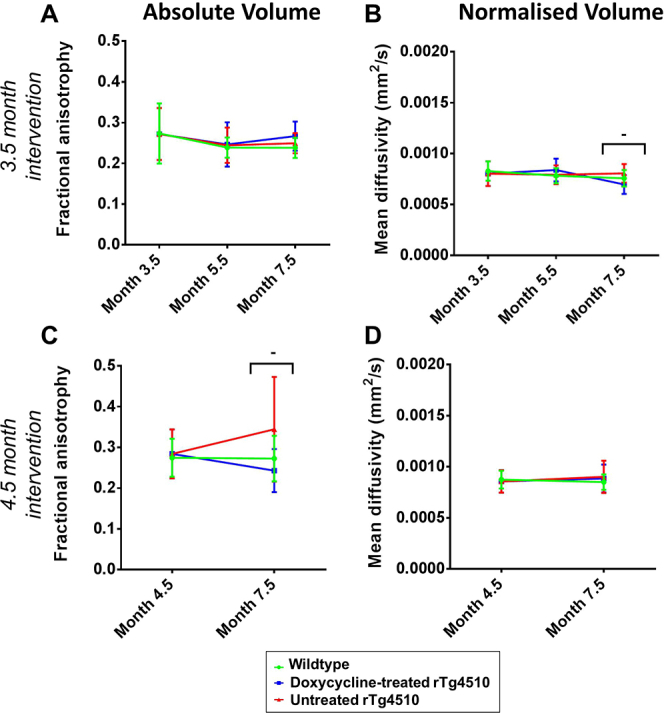
Longitudinal (A and C) fractional anisotropy and (B and D) mean diffusivity in the thalamus after (A and B) 3.5 months and (C and D) 4.5 months doxycycline intervention. Statistically significant groups have been identified and highlighted. Error bars represent the standard deviation. Treated rTg4510s versus untreated rTg4510s: −*p* ≤ .05.

After treatment with doxycycline from 3.5 months, we observed no significant difference to control values of MD in the treated rTg4510 mice at 7.5 months (Fig. 5B). In this instance, MD readily discriminates between the treated and untreated rTg4510 mice (*p* < .0001) and the untreated rTg4510 mice and wild-type controls (*p* < .0001) at 7.5 months. We identified a similar pattern of results within the 4.5-month intervention cohort; in particular, we observed good discrimination in FA between treated and untreated rTg4510 mice (*p* < .001) (Fig. 5C and D: 7.5 months). In contrast, the thalamus presented with comparatively few significant differences in DTI parameters between the 2 cohorts in both longitudinal studies (Supplementary Fig. 6).

**Fig. 6:**
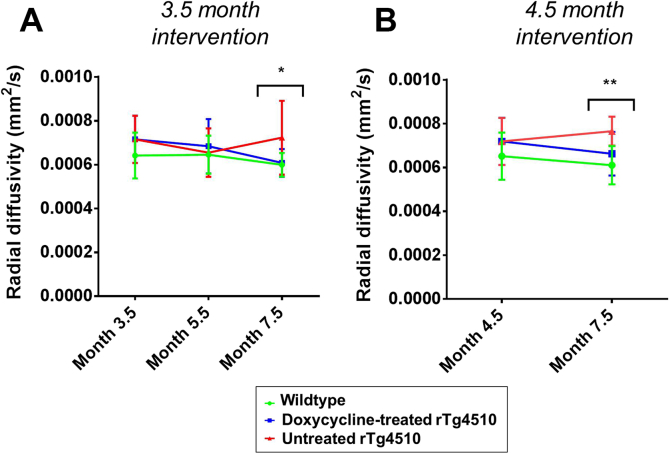
Longitudinal radial diffusivity in the corpus callosum after (A) 3.5 months and (B) 4.5 months doxycycline intervention. Statistically significant groups have been identified and highlighted. Error bars represent the standard deviation. Wild-type versus untreated rTg4510s: *p ≤ .05 and **p ≤ .01.

The RD results for the white matter region in the corpus callosum are shown in Fig. 6. Our findings of increased RD in the rTg4510 mice are consistent with the previous findings in this model (Sahara et al., 2014). The mean RD in the treated group was less than the untreated group at 7.5 months in both longitudinal studies and showed a trend toward wild-type values; however, these differences were nonsignificant (Fig. 6). Similarly to FA and MD (Fig. 5), RD is not able to distinguish between the rTg4510 mice and wild-type controls at earlier time points, further reinforcing our observations that the DTI parameters may not be sensitive to early neuropathology in this model. Taken together, the pattern of FA, MD, and RD results suggest that these DTI parameters are sensitive to more downstream events after suppression of the tau transgene.

### 3.5 Cerebral blood flow

**Supplementary Fig. 7:**
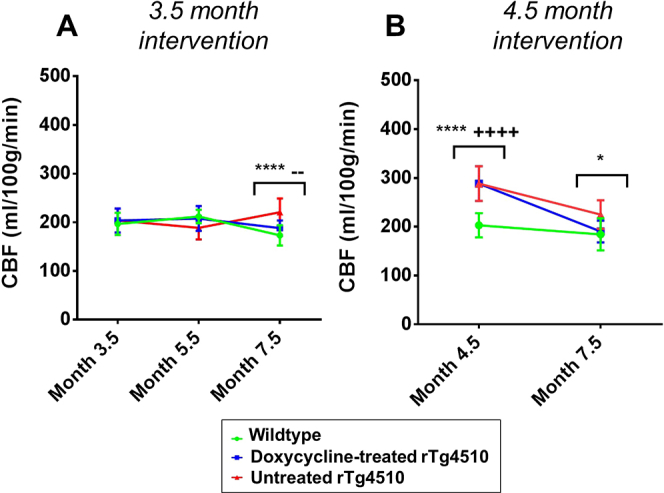
Longitudinal cerebral blood flow (CBF) results from the high-ranked pathology regions after (A) 3.5 months and (B) 4.5 months doxycycline intervention. Statistically significant groups have been identified and highlighted. Error bars represent the standard deviation. Wild-type versus untreated rTg4510s: *p ≤ .05 and ****p ≤ .0001. Wild-type versus treated rTg4510s: ++++*p* ≤ .0001. Treated rTg4510s versus untreated rTg4510s: −−*p* ≤ .01.

**Supplementary Fig. 8:**
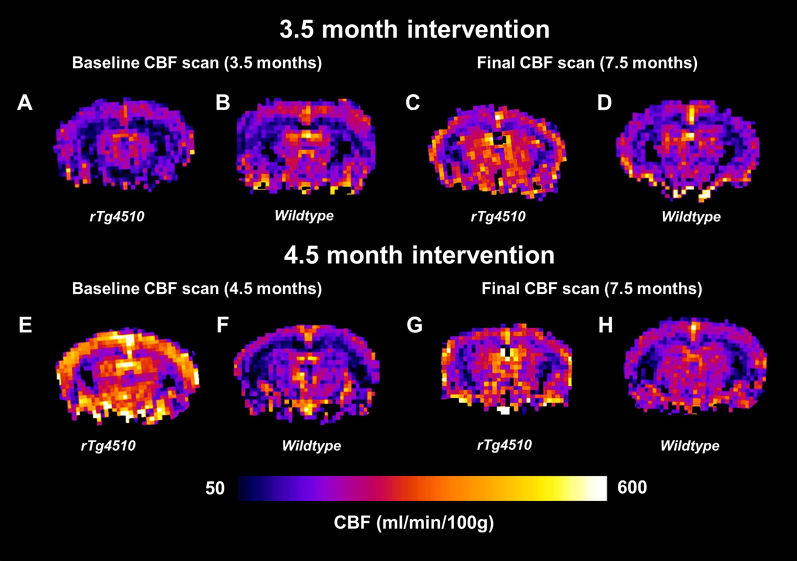
Cerebral blood flow (CBF) maps for representative wild-type and rTg4510 mice at the baseline scan (3.5 months for the earlier intervention study and 4.5 months for the later intervention study) and the final scan at 7.5 months.

We used ASL to measure CBF in the rTg4510 mice. The results within the high-ranked pathology regions, after 3.5 and 4.5 months doxycycline intervention, are shown in Supplementary Fig. 7. In the 4.5-month intervention study, we detected significant elevation in CBF in the rTg4510 mice compared with the wild-type controls (*p* < .0001) (Supplementary Fig. 7B: 4.5 and 7.5 months). Unexpectedly, this observation was not replicated in the 3.5-month intervention study (Supplementary Fig. 7A). Blood flow maps for representative rTg4510 mice and wild-type controls at baseline and final imaging time points are presented in Supplementary Fig. 8, to illustrate our findings of hyperperfusion in the rTg4510 mice at 4.5 months. This discrepancy may be because of subtle differences in the concentration of isoflurane, a vasodilator, between the 2 animal cohorts (1.5%) (Drummond et al, 1986, Matta et al, 1999).

**Supplementary Fig. 9:**
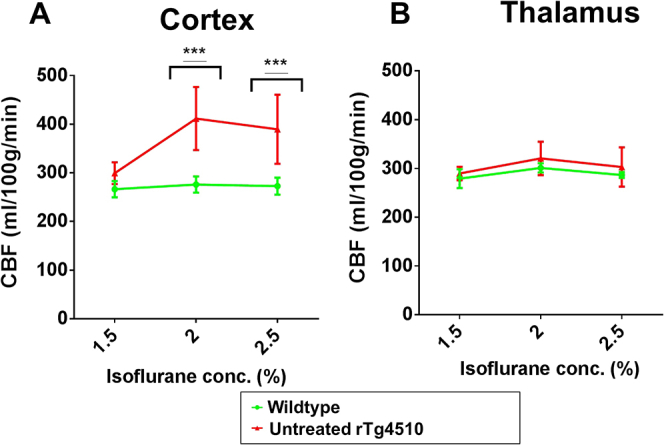
Cerebral blood flow (CBF) results within the (A) cortex and (B) thalamus at different concentrations of isoflurane. Statistically significant groups have been identified and highlighted. Error bars represent the standard deviation. Wild-type versus untreated rTg4510s: ***p ≤ .001.

We endeavored to maintain consistency in the experimental setup between imaging time points; this included executing the ASL sequence within the first 45 minutes of the mouse being anesthetized to reduce complications because of prolonged anesthesia. Despite our best efforts, we believe that miscalibration of the isoflurane vaporizer may have resulted in differences in the depth of anesthesia. To investigate this hypothesis further, we acquired CBF measurements at increasing concentrations of isoflurane in a separate cohort of aged rTg4510 mice (9 months) and wild-type controls. We observed a marked increase in cortical CBF (high tau region) changing from 1.5% to 2% isoflurane in the rTg4510 mice but not in the thalamus (low tau region) (Supplementary Fig. 9). This may indicate that high-ranked tau pathology regions may be more vulnerable to isoflurane-driven hyperperfusion; no significant increases in CBF were observed in the thalamus.

## 4 Discussion

In this work, we report the first application of multiparametric MRI biomarkers, including structural MRI, ASL, DTI, and APT, in a longitudinal study of neurodegeneration in the rTg4510 mouse model of tauopathy.

Histologic evaluation of pathologic tau revealed a distinct pattern of regional severity, with high deposition within the cortex and hippocampus, and milder affliction of the thalamus as previously observed (Wells et al., 2015). Treatment with doxycycline dramatically reduced the amount of PG-5 positive neurons in all the regions analyzed, through deactivation of the promoter within the transgene that drives tau expression and subsequent NFT formation (SantaCruz et al., 2005). Although observed in both treatment groups, the effect of doxycycline was more marked in the hippocampus and thalamus of the early (3.5 months) treatment group, suggesting that the transgene was repressed before the accumulation of significant tau pathology in these regions. These histologic results illustrate the efficacy of the doxycycline intervention for reducing tau accumulation. We sought to exploit the dynamic range in tau accumulation in rTg4510 mice treated with doxycycline from 3.5 and 4.5 months, and vehicle-treated rTg4510 mice, to assess the sensitivity of our MRI biomarkers to the development and suppression of pathologic tau.

Structural MRI is now routinely employed in large-scale longitudinal studies of AD patients such as the Alzheimer's Disease Neuroimaging Initiative, which aims to identify early biomarkers and evaluate emerging AD interventions (Jack et al., 2008). It has been hypothesized that MR-detectable structural changes may precede cognitive impairment in AD, supporting the clinical utility of this technique for early diagnosis. In this work, we used structural MRI to evaluate neurodegeneration in the rTg4510 mice, in conjunction with TBM and atlas-based parcellation. Both of these image processing techniques offer an unbiased estimation of brain atrophy and eliminate the need for time-consuming manual methods for structural data analysis (Ma et al., 2014).

Our TBM results identified gross structural changes within the rTg4510 brains, including extensive atrophy within the cortex. Gross morphometric changes in the later stage of the disease have previously been described in this model and substantiate our current imaging and histologic findings (Wells et al, 2015, Yang et al, 2011). These changes are markedly reduced after treatment with doxycycline, suggesting that structural MRI is sensitive to the suppression of pathologic tau in this model. The atlas-based parcellation results provided quantitative regional volume estimates, with detectable atrophy occurring within the high-ranked pathology regions of the rTg4510 animals from 3.5 months. However, this volume difference was also seen in the thalamus (low-ranked pathology), indicating that this difference could be because of either neurodevelopmental effects of transgene expression during prenatal development or brain alterations and compensation in response to the forebrain atrophy. Normalizing the absolute volumes to total brain volume revealed a pattern of atrophy that is specific to tau pathology and accounts for the early global changes in brain volume. The normalized volume data revealed deterioration of the high-ranked pathology regions detectable at 5.5 months, proceeding the development of NFTs within the cortex from 4 months (SantaCruz et al., 2005). We also observed a reduction in atrophy within rTg4510 mice treated with doxycycline; this was more apparent when treatment began at the earlier time point of 3.5 months. Normalizing the absolute volumes to total intracranial volume instead of total brain volume may mitigate the proportional increases observed within tissues unaffected by tau pathology.

Our results suggest that the presence of pathologic tau underpins the key structural changes seen in this model. In addition to detectable volume losses within areas expressing high tau burden, the suppression of the tau transgene resulted in markedly reduced atrophy, which correlates with the reduction of PG-5–positive cells in the high-ranked pathology regions.

Our findings support the application of structural MRI as a close correlate of tau pathology, where it has already garnered clinical acceptance (Jack et al., 2002). In addition, we believe that structural MRI may serve as a valuable biomarker for assessing therapies targeting the production or clearance of pathologic tau and the associated neurodegeneration and volume loss.

APT is a comparatively new technique for measuring the exchange of bulk water protons with amide protons of mobile cellular protein (Zhou et al, 2003a, Zhou et al, 2003b). APT is more commonly used for imaging brain tumors (Jones et al, 2006, Zhou et al, 2003a, Zhou et al, 2003b) owing to its sensitivity to changes in protein expression in malignant tissues (Zhou et al., 2008). In this work, we observed a reduction in APT in high-ranked pathology regions within the rTg4510 mice compared with wild-type controls at 7.5 months. These changes may be caused by the presence of neuronal tau pathology, impairing the chemical exchange between the amide protons and water protons (Wells et al., 2015). No significant differences were observed within the thalamus, where tau pathology was notably mild, further supporting this interpretation.

Although APT aims to provide a direct measure of proton exchange, it is highly sensitive to changes in tissue pH. Acidification of the brain of AD patients has already been observed (Fang et al., 2010) and attributed to the accumulation of acidic metabolites and a breakdown in the brain's ability to maintain a constant internal pH. The decreased APT within the rTg4510 mice may, therefore, also reflect changes in tissue pH within the high-ranked pathology regions. Isoflurane anesthesia is not believed to alter tissue pH (Constantinides et al., 2011, Makaryus et al, 2011), although the implications after prolonged anesthesia have yet to be explored. To mediate any isoflurane-related changes in tissue pH, the CEST acquisition was performed at the beginning of the imaging protocol.

In addition to pH, the APT signal can also be influenced by the cellular water content and the T1 relaxation time of the bulk tissue water (McVicar et al., 2014). It is also worth noting that APT is highly dependent on B0 field homogeneity (Scheidegger et al., 2011), especially when applied to in vivo imaging data at high magnetic field strengths (Sun et al., 2007); in this work, the influence of B0 field inhomogeneities was alleviated by separately shimming over the CEST image acquisition volume before image acquisition. It is difficult to elucidate one underlying factor that is causing the APT changes in this model; regardless, our results illustrate the sensitivity of measures of APT to tau-related pathology in this model and may reflect neurodegenerative processes. In particular, it may serve as an important biomarker when assessing the efficacy of novel therapeutics targeting pH-sensitive pathways, such as neuronal transmitters (Anand et al., 2014).

DTI is increasingly being employed in longitudinal studies of AD (Acosta-Cabronero et al, 2012, Mielke and et al, 2009). It can offer heightened sensitivity to changes in the gray and white matter, which are not so readily detectable using structural MRI. It is believed that DTI changes may precede volume changes in AD and, therefore, may represent an important early biomarker of the disease with enhanced sensitivity to early microstructural changes (Hugenschmidt et al., 2008).

RD is a valuable DTI parameter that reflects diffusion perpendicular to the primary direction in a voxel, which may reflect fiber orientation in the white matter. Our observation of increased RD in the corpus callosum mimics the clinical picture (Huang et al., 2012) and has been previously observed in the rTg4510 mouse (Wells et al., 2015). Demyelination has been cited as a possible mechanism underpinning this change (Sahara et al, 2014, Song et al, 2004). This is supported by the previous work in the rTg4510 mouse, where electron microscopy revealed swollen unmyelinated processes in the corpus callosum from 4 months (Sahara et al., 2014). No axial diffusivity changes have been observed in this model, suggesting that demyelination is occurring in the absence of axonal injury (Song et al., 2002). Alterations in RD were not significantly different until the final imaging time point at 7.5 months; our observations, therefore, suggest that RD may be sensitive to downstream events proceeding the accumulation of pathologic tau and unmyelinated processes from 4 months (SantaCruz et al., 2005). Treatment with doxycycline marginally reduced the RD changes; however, there was no marked improvement in this parameter when treatment was commenced earlier.

Although DTI is traditionally used to characterize white matter structures, it can also be applied to fiber-rich gray matter structures such as the hippocampus (Qin et al., 2013). We used FA and MD within the high-ranked pathology regions of the cortex and hippocampus. Our finding of increased FA within the rTg4510 mice was unexpected, as this traditionally implies more intact axons. However, the phenomenon of increased FA in brain regions undergoing gross degenerative processes has previously been observed (Kerbler et al., 2013) and may suggest increased disruption and loss of isotropic cells (Kerbler et al., 2013). Meanwhile, our observation of increased MD in the rTg4510 mice mimics the clinical condition (Cherubini et al, 2010, Kantarci et al, 2001); this change is believed to indicate enlargement of the extracellular space, suggesting neurodegeneration is occurring (Cherubini et al, 2010, Sykova, 2004).

Similarly to the RD results, FA and MD in the high-ranked pathology regions were only able to discriminate between the rTg4510 mice and wild-type controls at the final imaging time point. However, these parameters offered heightened sensitivity to the doxycycline treatment. After 3.5 and 4.5 months doxycycline intervention, we identified recovery of both FA and MD, suggesting that the presence of pathologic tau is underpinning these DTI changes. We observed greater recovery of MD after earlier (3.5 months) treatment with doxycycline, suggesting that MD may be more sensitive to suppression of pathologic tau.

Although it has been claimed that microstructural changes detectable using DTI may precede structural changes (den Heijer et al., 2012), our results do not support this hypothesis for this model. Increased FA, MD, and RD in the rTg4510 mouse were only detectable at 7.5 months, in the presence of significant atrophy and tau burden at this time point. We, therefore, propose that the strength of DTI may not lie in its ability to discriminate between rTg4510 mice and wild-type controls; rather, it offers valuable information that may compliment the structural MRI results. This may be particularly beneficial when assessing therapeutic efficacy of treatments that target pathways for which DTI offers heightened sensitivity.

There is growing evidence supporting the role of CBF as a biomarker of AD (Wolk and Detre, 2012). The vascular hypothesis for AD stipulates that underlying vascular factors such as hypertension, diabetes, and obesity may substantially contribute to the development of AD pathogenesis (Ruitenberg et al, 2005, de la Torre, 2004). Measurements of CBF, therefore, may represent an important biomarker in the early diagnosis of AD and for evaluation of therapies that may produce a vascular response (Hajjar and Rodgers, 2013). CBF differences have already been observed in AD patients using ASL, with decreased perfusion in cortical regions affected by the disease (Wolk and Detre, 2012).

Previous observations in the rTg4510 mouse identified a distinct pattern of hyperperfusion in the cortical and hippocampal regions in an advanced stage of the pathology (Wells et al., 2015). In the 4.5-month intervention study, we noted distinct hyperperfusion consistent with this previous work, although this was not replicated in the 3.5-month intervention study. To understand this further, we investigated the effects of the anesthetic isoflurane in the rTg4510 mouse. We hypothesized that these discrepancies may be because of subtle differences in the concentration of administered isoflurane; a volatile anesthetic that has been shown to cause a dose-dependent increases in CBF (Matta et al., 1999). This theory was supported by evidence of hyperperfusion at high concentrations of isoflurane (Drummond et al., 1986). Interestingly, we noted that when the mice were challenged with a higher concentration of isoflurane, the regions of high tau pathology responded with a marked increase in CBF, which was much less evident in the wild-type controls. Although it is unclear why this is the case, a recent work demonstrates that the rTg4510 mouse has increased cerebrovascular response (Wells et al., 2014), which could suggest that elevated levels of blood CO_2,_ because of the reduced respiration rate that accompanies increased delivery of isoflurane, may result in the increased CBF. Similar results have recently been observed in a bigenic mouse model of AD, where increased cerebrovascular response in response to hypoventilation was reported (Govaerts et al., 2014). Furthermore, the sensitivity of the rTg450 mouse to increased isoflurane concentration may be because of a breakdown in autoregulation (Gozzi et al., 2007), a mechanism that may be affected as a result of tau pathology, leading to vascular compensation and the increase in CBF, although the precise mechanism is still unknown (Wells et al., 2014).

A number of additional rodent studies have reported cortical hypoperfusion using ASL in conjunction with isoflurane anesthesia at varying concentrations: 1%–1.5% (Faure et al., 2011), 1.5%–2% (Massaad et al., 2010), and 2% (Weidensteiner et al., 2009). However, these studies all focus on models exhibiting amyloid pathology. Our observations of hyperperfusion at high concentrations of isoflurane may be unique to the rTg4510 mouse and reflect a tau-related effect. However, clinical blood flow studies have reported that the vasodilating properties of isoflurane result in redistribution of regional CBF measurements, which must be taken into account when interpreting our findings in the rTg4510 mouse (Villa et al., 2012). Despite the complexity surrounding CBF measurements, at higher concentrations of isoflurane, we observed complete discrimination of the rTg4510 mice from wild-type controls, suggesting it may be a useful biomarker for evaluating the rTg4510 mouse's response to candidate therapies. However, careful calibration of administered anesthetic gases is required in future studies to extract meaningful longitudinal CBF measurements in the rTg4510 mouse.

Previous work in the rTg4510 mouse at 8.5 months indicated that hyperperfusion and increased MD were detectable in regions of low tau pathology (the thalamus of the rTg4510 [mean NFT density = 2.3 cells/mm^2^]) (Wells et al., 2015). Thus, we considered these biomarkers to be strong candidates for relatively early detection of tau-driven abnormalities in the cortex and hippocampus of the rTg4510. However, in this work, no differences in MD between the WT and rTg4510 cohorts were detected in the high-rank regions at 3.5, 4.5, or 5.5 months. Increased CBF was observed in the rTg4510 mice at baseline in the 4.5-month intervention study, but this finding was not reproduced at 3.5 or 5.5 months in the 3.5-month intervention study, likely owing to discrepancies in anesthetic delivery between the 2 longitudinal studies.

Finally, it should be noted that this is a mouse model of tauopathy, which develops robust NFT pathology and AD-related neuropathologic changes in the absence of amyloid plaques. In this work, we uncovered tau-specific alterations, as this model permits the dissection of tau's role in the neurodegenerative cascade. However, caution must be taken when interpreting these findings in the context of the clinical condition, in the presence of both plaques and NFTs.

## 5 Conclusions

This study demonstrates the value of non-invasive multiparametric quantitative MRI for longitudinal assessment of tau pathology in the rTg4510 model and monitoring distinct properties that reflect the biological responses to therapy in AD. Our imaging protocol permits the acquisition of structural, ASL, DTI, and APT data in a longitudinal framework within the 3-hour anesthesia tolerance of mice (Lerch et al., 2012). The non-invasive nature of ASL, DTI, and APT data acquisitions potentially permits relative ease of translation to the clinical setting. However, further standardization and refinement are required to provide useful diagnostic data in a clinically feasible imaging time.

These diverse scans all offer complimentary information and provide insight into different pathologic mechanisms occurring within the disease process. The structural scans and analysis proved to be the earliest biomarker, in addition to offering the highest degree of sensitivity to the doxycycline treatment; however, over half of the imaging time is dedicated to the acquisition of the structural data, which should be considered on interpretation.

In summary, these data represent a platform for future longitudinal and therapeutic efficacy studies of novel therapeutic strategies that target varying aspects of the pathology time course in this model.

## Disclosure statement

The authors have no conflicts of interest to disclose.
